# Microglial Activation in the Retina of a Triple-Transgenic Alzheimer’s Disease Mouse Model (3xTg-AD)

**DOI:** 10.3390/ijms21030816

**Published:** 2020-01-27

**Authors:** Elena Salobrar-García, Ana C. Rodrigues-Neves, Ana I. Ramírez, Rosa de Hoz, José A. Fernández-Albarral, Inés López-Cuenca, José M. Ramírez, António F. Ambrósio, Juan J. Salazar

**Affiliations:** 1 Instituto de Investigaciones Oftalmológicas Ramón Castroviejo, Universidad Complutense de Madrid, 28040 Madrid, Spain; elenasalobrar@med.ucm.es (E.S.-G.); airamirez@med.ucm.es (A.I.R.); rdehoz@med.ucm.es (R.d.H.); joseaf08@ucm.es (J.A.F.-A.); inelopez@ucm.es (I.L.-C.); ramirezs@med.ucm.es (J.M.R.); 2 Coimbra Institute for Clinical and Biomedical Research (iCBR), University of Coimbra, 3000-548 Coimbra, Portugal; catarinaneves.fctuc@gmail.com (A.C.R.-N.); afambrosio@fmed.uc.pt (A.F.A.); 3 Center for innovative Biomedicine and Biotechnology (CIBB), University of Coimbra, 3004-517 Coimbra, Portugal; 4 Fac. Óptica y Optometría, Universidad Complutense de Madrid, 28037 Madrid, Spain; 5 Fac. Medicina, Departamento de Oftalmología. Universidad Complutense de Madrid, 20040 Madrid, Spain; 6 AIBILI-Association for Innovation and Biomedical Research on Light and Image, 3000-548 Coimbra, Portugal

**Keywords:** Alzheimer’s disease, retina, neuroinflammation, microglia, triple transgenic Alzheimer’s disease mouse model, 3xTg-AD, morphometric analysis

## Abstract

Alzheimer’s disease (AD) is the most common type of dementia in the world. The main biomarkers associated with AD are protein amyloid-β (Aβ) plaques and protein tau neurofibrillary tangles, which are responsible for brain neuroinflammation mediated by microglial cells. Increasing evidence has shown that the retina can also be affected in AD, presenting some molecular and cellular changes in the brain, such as microglia activation. However, there are only a few studies assessing such changes in the retinal microglia in animal models of AD. These studies use retinal sections, which have some limitations. In this study, we performed, for the first time in a triple-transgenic AD mouse model (3xTg-AD), a quantitative morphometric analysis of microglia activation (using the anti-Iba-1 antibody) in retinal whole-mounts, allowing visualization of the entire microglial cell, as well as its localization along the extension of the retina in different layers. Compared to age-matched animals, the retina of 3xTg-AD mice presents a higher number of microglial cells and a thicker microglial cell body area. Moreover, the microglia migrate, reorient, and retract their processes, changing their localization from a parallel to a perpendicular position relative to the retinal surface. These findings demonstrate clear microglia remodeling in the retina of 3xTg-AD mice.

## 1. Introduction

Alzheimer’s disease (AD) is a progressive brain disorder that represents the most common cause of dementia among the elderly [1] and is characterized by a loss of neurons and their synapses in the cerebral cortex [2]. The principal hallmarks of AD are the extracellular deposit of the protein amyloid-β (Aβ), which forms plaques, and the intraneuronal accumulation of the hyper-phosphorylated microtubule-associated protein tau, which forms neurofibrillary tangles [3]. These deposits, principally located in the cortex, hippocampus, and amygdala, can induce neuronal death by apoptosis [4].

Previous studies have demonstrated the existence of a neuroinflammatory component in AD, including astroglial and microglial activation, an increase in the expression of inflammatory factors, and activation of complementary systems (both classic and alternative pathways) [5,6]. Neuroinflammation can be related to Aβ deposits, which produce a strong inflammatory response and alter the microglial phagocytosis of Aβ deposits [7]. These phenomena can maintain the overexpression of inflammatory mediators, which can induce neuronal death [8]. It is clear that microglia activation can contribute to the progression of AD, so strategies for controlling microglia activation could have beneficial effects in slowing the progression of this disease [9].

In AD patients, alterations in the retinal structure detected by Optical Coherence Tomography (OCT) have been reported, suggesting that these alterations could be useful as biomarkers [10,11,12]. According to these studies, in the retina of AD patients, as well as in human postmortem specimens, Aβ plaques have been found from the outer plexiform layer (OPL) to the nerve fiber layer (NFL) [13,14,15,16,17]. Aβ accumulation in the retina can contribute to retinal neurodegeneration and thus lead to the loss of the retinal ganglion cells observed in AD human retinas [16,18,19]. In addition, γ-synuclein, a highly expressed protein in retinal ganglion cells, changes its expression in the retina of patients with AD [20,21]. In AD transgenic mouse models (APPswe/PS11E9, Tg2576AD, 3xTg-AD, PSAPP, and 5xFAD), researchers have also observed the presence of Aβ plaques in the different retinal layers (NFL, ganglion cell layer (GCL), inner plexiform layer (IPL), and outer plexiform layer (OPL)) [22,23,24,25,26,27,28]. 

As previously stated about inflammation in the AD brain, microglial activation has been found to be related to Aβ plaques [29]. In the presence of an insult, the microglia—resident innate immune cells of the central nervous system—adopt an activated stage, change their shape (shortening their processes and enlarging their soma), proliferate, and migrate, thereby accumulating around the damaged areas. In the highest activation stage, the microglia acquire an amoeboid morphology and actuate as macrophages [30]. In association with morphological changes, the expression of enzymes, receptors, and release of inflammatory factors are altered in microglia [30]. Excessive microglial activation could cause the release of neurotoxic factors, inducing neuronal death [31]. However, the studies that have analyzed AD microglial activation in the retina are very scarce and were performed using different AD mouse models [16,27,32,33,34]. Only one study carried out in retinal sections examined microglial cells in 3xTg-AD mice that show many of the main features of AD, since these mice have mutations present in human genes encoding Tau (MAPTP301L), presenilin 1 (PS1M146V), and amyloid precursor protein (APPSwe) [27].

In the present work, we performed a quantitative and morphological evaluation of the microglial cells present in the retina of 3xTg-AD mice. For this process, we used retinal whole-mounts to facilitate visualization of the entire microglial cells and their localization along the extension of the retina (on the X, Y, and Z-axes). This analysis provides accurate morphological data that will help us understand how these cells change along the different layers of the retina. We also quantified the number and soma size of these microglial cells in 3xTg-AD mice and in age-matched controls.

## 2. Results

### 2.1. Qualitative Analysis of Iba-1^+^ Cells 

**Figure 1:**
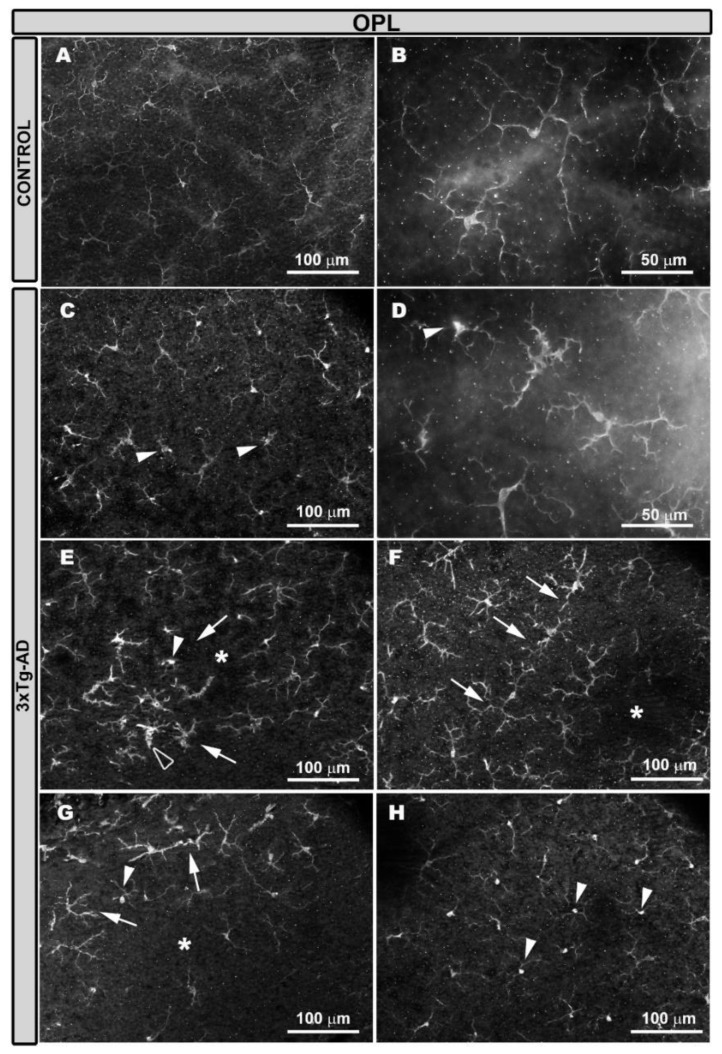
Iba-1^+^ cells in the outer plexiform layer (OPL). The retinal whole-mount labelled with anti-Iba-1. In the age-matched control animals’ eyes (**A**,**B**), Iba1^+^ cells have a ramified morphology and constitute a regular plexus of tiled cells throughout the retina. In 3xTg-AD animals (**C**–**H**), soma thickening and process retraction were observed in the Iba-1^+^ cells (**C**,**D**). The mosaic-like structure had disappeared in some areas of the retina (**D**–**G**) because there were areas of cell grouping (arrows) at the expense of areas without cells (*). These groups of cells can be circular (**E**) or oriented in rows (**F**,**G**); cells perpendicular to the retinal surface (the white arrowhead in **E** and **G**) and amoeboid cells (hollow arrowhead in **E**) can also be observed. Iba-1^+^ perpendicular cells in some areas of the retina occupied large zones (the white arrowhead in **H**).

**Figure 2:**
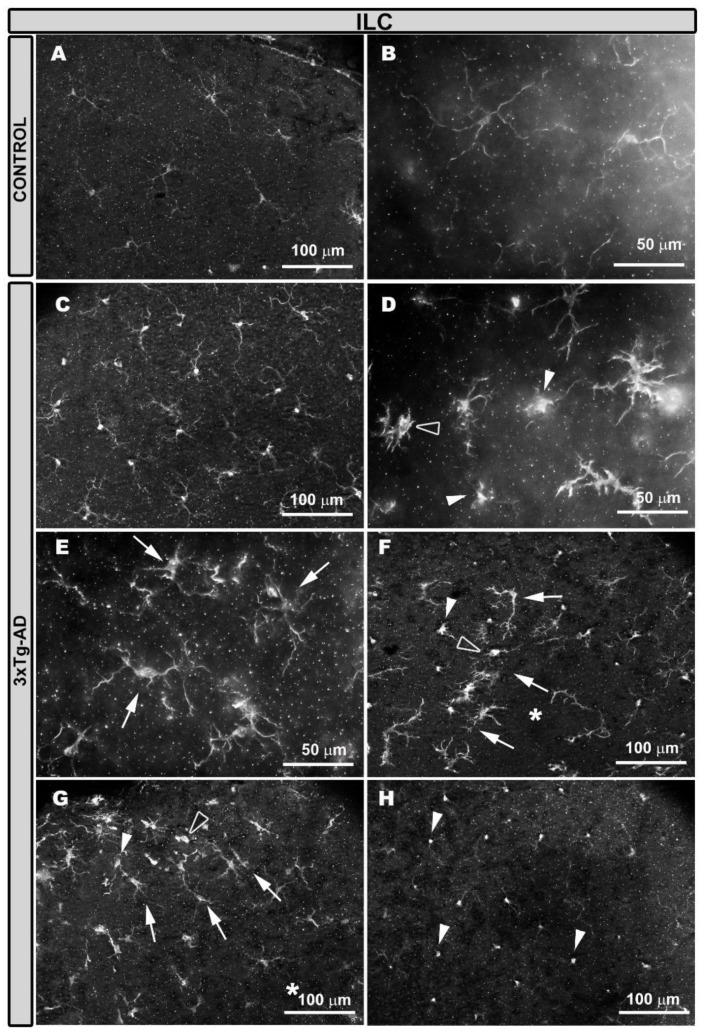
Iba-1^+^ cells in the inner retinal layer complex (ILC). Retinal whole-mount labelled with anti-Iba-1. In the age-matched control animals’ eyes (**A**,**B**), most of the Iba-1^+^ cells had a ramified appearance and formed a regular mosaic-like plexus through the retina. In 3xTg-AD animals (**C**–**H**), the Iba-1^+^ cells thickened, and their processes were retracted (**C**,**D**). In these retinal layers, the mosaic-like plexus was not homogeneous (**D**–**G**), and there were zones of cellular grouping (arrows) in a circular shape (**E**) or in rows (**F**,**G**). Areas without cells were also found (*). In the areas of cell grouping, amoeboid type cells (hollow arrowhead) and vertically arranged cells (white arrowhead) were observed; the latter were also found in other areas of the retina, forming large extensions (white arrowhead in **H**). The ILC is constituted by the inner plexiform layer and the nerve fiber layer–ganglion cell layer.

In age-matched wild type (WT) mice, Iba-1^+^ cells were located along the extension of the retina in a regular plexus of tiled cells, with an equidistant-like separation among them in different retinal layers: OPL (Figure 1A) and the inner retinal layer complex (ILC), constituted by IPL and GCL-NFL (Figure 2A). Iba-1^+^ cells showed an ovoid soma and a ramified morphology, with primary, secondary, and tertiary processes located parallel to the retinal surface (Figure 1A,B and Figure 2A,B). In 3xTg-AD animals, Iba-1^+^ cells exhibited a thickening of the soma and a retraction of the cell processes (Figure 1C,D and Figure 2C,D). As in WT animals, Iba-1^+^ cells were distributed along the retinas in the OPL and ILC but not in a regular manner (Figure 1C–G and Figure 2C–G). The mosaic-like plexuses of Iba-1^+^ cells disappeared in some zones of the retinal layers because Iba-1^+^ cells were grouped in some areas and separated in others (Figure 1E–G and Figure 2E–G). There were two groups of Iba-1^+^ cells: One formed circular areas and oriented their processes towards the center of the circle (Figure 1E and Figure 2E), while the other formed rows (Figure 1F,G and Figure 2F,G), and the nearest Iba-1^+^ cells reoriented their processes toward them. The grouped Iba-1^+^ cells showed a greater retraction of the cell processes and thicker somas than the rest of the Iba-1^+^ cells, sometimes acquiring an amoeboid appearance (Figure 1E and Figure 2F,G), and the other cells were arranged perpendicularly (Figure 1E and Figure 2F,G).

The loss of the mosaic-type structure was also due to a change in the orientation of some Iba-1^+^ cells from parallel to perpendicular to the retinal surface. This perpendicular orientation caused the cells to occupy less space in the plexus (Figure 1H and Figure 2H). 

**Figure 3:**
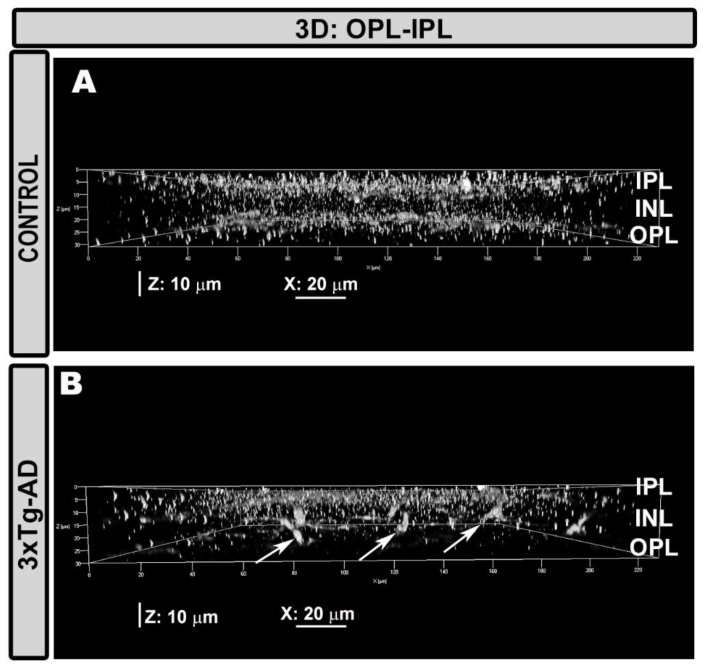
Iba-1^+^ cells among the IPL–OPL. Retinal whole-mount. Iba-1 immunostaining. Cut-view analysis on the YZ plane. In some retinal areas of 3xTg-AD mice (**B**), some Iba-1^+^ cells were arranged perpendicular to the retinal plane (arrow) and were observed to occupy the IPL, INL, and OPL. This was not seen in the age-matched control group (**A**).

The perpendicular Iba-1^+^ cells located their somas in nuclear retinal layers and extended their processes to the nearest Iba-1^+^ plexuses, thereby connecting with them, as observed using the 3D view tool, which provides cross-sectional images of the retina (Figure 3). These cells showed an increment of Iba-1^+^ staining in the soma compared with the cells located parallel to the retinal plane (Figure 1H and Figure 2H). 

### 2.2. Quantitative Analysis of Iba-1^+^ Cells 

#### 2.2.1. Number of Iba-1^+^ Cells in OPL and ILC

**Figure 4:**
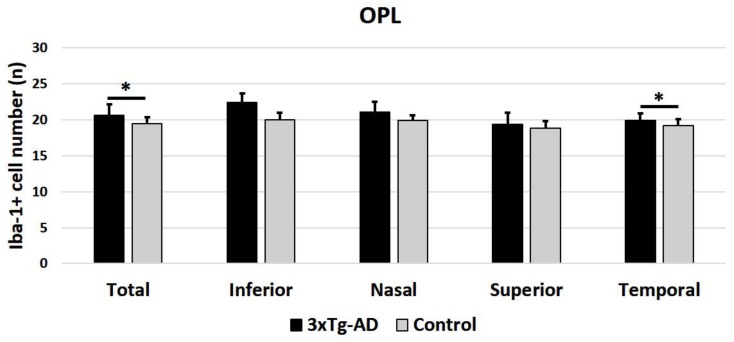
Number of Iba-1^+^ cells in the outer plexiform layer (OPL) in the 3xTg-AD and in age-matched control mice in the different retinal zones (inferior, nasal, superior, and temporal), as well as in the total retina. A total of 192 images were analyzed for each study group (3xTg-AD and control). Histograms show the mean number (± standard deviation, SD) of the Iba-1^+^ cells per area of 0.1502 mm^2^. Statistically significance indicators: * *p* < 0.05.

**Figure 5:**
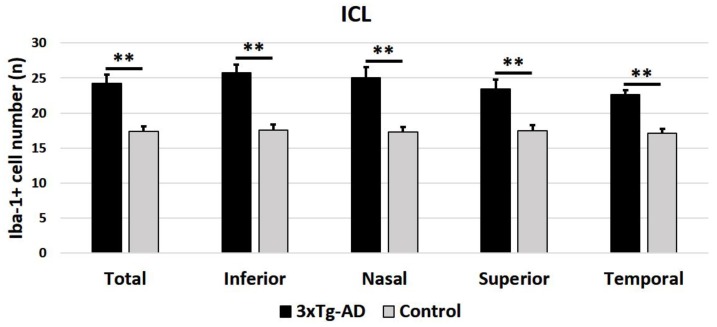
Number of Iba-1^+^ cells in the inner retinal layer complex (ILC) in the 3xTg-AD and in age-matched control mice in the different retinal zones (inferior, nasal, superior, and temporal), as well as in the total retina. A total of 192 images were analyzed for each study group (3xTg-AD and control). Histograms show the mean number (± standard deviation, SD) of Iba-1^+^ cells per area of 0.1502 mm^2^. Statistically significance indicators: ** *p* < 0.01.

When the analysis was performed considering the means of all retinal areas, the number of Iba-1^+^ cells was significantly higher in the 3xTg-AD eyes than in the control eyes, both in the OPL (*p* < 0.05) (Figure 4) and in the ILC (*p* < 0.001) (Figure 5). The sectorial retinal analysis of the number of Iba-1^+^ cell demonstrated that in the OPL, only the inferior quadrant of the 3xTg-AD eyes showed a significant increase compared to the control eyes (*p* < 0.05). In the ILC of transgenic mice, all quadrants (superior, inferior, nasal, and temporal) exhibited a significant increase in their Iba-1+cell number with respect to the control (*p* < 0.001 in all instances).

#### 2.2.2. Cell Body Area of Iba-1^+^ Cells in the OPL and in the ILC

**Figure 6:**
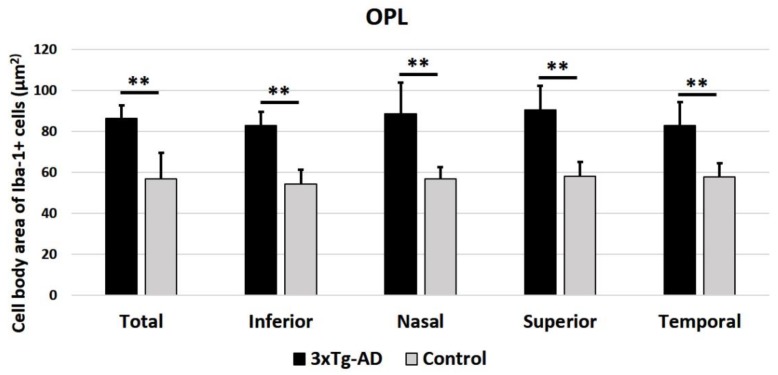
Cell body area of Iba-1^+^ cells in the outer plexiform layer (OPL), in 3xTg-AD, and in age-matched control mice in the different retinal zones (inferior, nasal, superior, and temporal), as well as in the total retina. A total of 576 microglial cells were measured for each study group (3xTg-AD and control). Histograms show the mean cell body area (± standard deviation, SD) of the Iba-1^+^ cells. Statistically significance indicators: ** *p* < 0.01.

**Figure 7:**
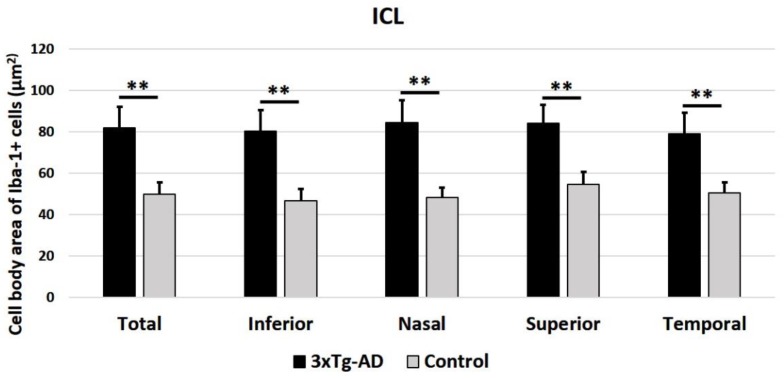
Cell body area of Iba-1^+^ cells in the inner retinal layer complex (ILC), in 3xTg-AD, and in age-matched control mice in the different retinal zones (inferior, nasal, superior, and temporal), as well as in the total retina. A total of 576 microglial cells were measured for each study group (3xTg-AD and control). Histograms show the mean cell body area (± standard deviation, SD) of Iba-1^+^ cells. Statistically significance indicators: ** *p* < 0.01.

In the OPL (Figure 6) and in the ILC (Figure 7) of 3xTg-AD eyes, the cell body area of Iba-1^+^ cells was significantly thicker compared to the control eyes, both for the mean of all retinal areas and by quadrant (superior, inferior, nasal, and temporal) (*p* < 0.001 in all instances).

## 3. Discussion

In this work, we demonstrated for the first time in retinal whole mounts of a transgenic triple mouse 3xTg-AD that retinal microglial cells (Iba-1^+^ cells) show signs of activation in the different retinal sectors (superior, inferior, nasal, and temporal) of all retinal layers (OPL, IPL, and GCL/NFL) where these cells are found. The alterations detected in Iba-1^+^ cells include (i) an increased cell body area, (ii) the retraction and reorientation of processes, (iii) the appearance of amoeboid cells, (iv) the radial disposition of the cell body, and (v) an increase in cell number and (vi) cell migrations, which produced alterations in the mosaic type plexuses of the Iba-1^+^ cells.

In AD, microglial activation in the brain has been demonstrated, indicating that a neuroinflammatory process occurs in this disease [5,35]. In a normal adult nervous system, microglia are found in a quiescent state characterized by a ramified morphology [36]. When damage occurs in the central nervous system, a series of changes appear in the extracellular environment, such as the presence of various molecules or molecules in non-physiological concentrations (i.e., cytokines and ATP) (on-signal), as well as the lack of some molecules that are released during normal neuronal activity as neurotransmitters (off-signal) [37]. These signals are detected by the microglial cells, and they become activated, and can transform into a macrophage-like morphology, known as an amoeboid microglia, which is characterized by the absence of cellular processes [38,39]. In AD, microglia activation may be related to Aβ deposits and neurodegeneration [7]. Most AD studies have been conducted on the brain [7,9,16,28,40,41]. Nevertheless, very few studies have focused on microglial activation in the retina using experimental models of AD [23,27,32,33,34,42].

In Tg2576AD transgenic mice, a significant increase in the number of retinal microglia was found compared to the controls using retinal sections labeled with anti-Iba-1 [34]. In APPswe/PS11E9 double transgenic mice, microglial activation was observed, in terms of the increased relative optical density in F4/80 immunoreactivity (used to identify macrophages and microglia), both in 12 and 16 month old mice (middle-aged) and in mice aged 19–21 months (elderly) [33]. In the same model, Ning et al. (2008) quantified the profile of ganglion cells surrounded by F4/80 microglial cells, demonstrating a significant increase in the number of transgenic animals at 27 months and postulating microglia activation [28].

The 3xTg-AD transgenic triple mouse model presents several features found in AD patients, thus constituting a suitable model for the study of this pathology [23]. In this model, Grimaldi et al. (2018) [27] assessed the microglial cells in retinal sections and quantified the number of Iba1^+^ microglial cells per volume (mm^3^), as well as the soma area and the extent of microglia ramification [27]. The authors found that at the pre-symptomatic AD stage ((5–10–20 post-natal weeks (PNWs)), microglial Iba-1^+^ cells were more ramified than the microglia present in WT mice. Nevertheless, at the late-symptomatic AD stage (50–72 PNWs), microglial cells displayed a less ramified morphology and were increased in number. Moreover, the authors found no differences in the microglial soma area. In this study, we also found signs of microglia activation in 3xTg-AD during the late symptomatic AD stage (64 PNWs). However, our study was carried out using retinal whole mounts, unlike previous studies on retinal microglia in AD transgenic animals, which were performed on retinal sections. These retinal sections do not allow one to visualize all the cells of the same layer in their entirety without missing a piece of the soma or some processes. In contrast, in retinal whole-mounts, cells are not sectioned, which allows the complete visualization of the microglial cell and its location along the extension of the retina (on the *X*, *Y*, and *Z* axes), thereby providing more precise data on its morphology and distribution (cell shape, soma size, and cell migration, among others). An accurate quantification of the number of microglial cells in each retinal layer can also be performed. In our 3xTg-AD animals, microglial cells showed a retraction of the processes and a significant increase in the area of the cell body in the different sectors of the retina (superior, inferior, temporal, and nasal) of all retinal layers where the microglia were located (OPL and IPL/NFL-GCL). In contrast to our study, Grimaldi et al. (2018) did not find differences in the soma’s size area [27]. This discrepancy may be because these authors used retinal sections and not retinal whole-mounts. In addition, in 3xTg-AD mice, we observed a significant increase in the total number of Iba-1^+^ cells in all retinal sectors of the ILC (IPL and GCL-NFL) and in the inferior sector of the OPL compared with the age-matched animals. Most of the studies using AD transgenic models counted the number of microglial cells indirectly [28,33,34], without providing data on the cell number increase depending on the layer and the retinal sector. The increase in microglial cell number could be due to microglial proliferation as an activation mechanism in response to neuronal damage, triggered by amyloid plaques [43] and/or by the neuroinflammation triggered by these deposits [5]. In the rat AD model, TgF344-AD [42], and APPswe/PS11E9 double transgenic mice [33], Aβ deposits were mainly situated in the plexiform layers. Although, in the APPswe/PS11E9 model, Ning et al. (2008) found deposits mostly in the GCL /NFL [28]. In the Tg2576AD transgenic mice, Aβ deposits were detected mainly in GCL and IPL, with some plaques found in the OPL and in the photoreceptor layer [34]. In the 3xTg-AD mouse model, other authors have detected Aβ deposits and pTau neurofibrillary tangles, which begin to appear in the retinal GCL during the pre-symptomatic stages of AD and are also detected in the outer layers during the disease’s progression. These deposits increase their volume during the late-symptomatic AD stage [27]. 

In our 3xTg-AD transgenic mice, the microglial arrangement in a mosaic-type plexus, typical of age-matched WT animals, was lost in some retinal areas because the cells were grouped in some areas, leaving others with lower cell density. The grouped cells formed rows or circular areas, and, in the latter, the microglial cells redirected their processes into the circle. The processes of these grouped microglial cells were retracted, and some amoeboid cells were observed among them. In the brains of AD patients, activated microglia surrounding Aβ plaques have been observed to likely contribute to the cleaning of these deposits [40]. In the retinal sections of the 3xTg-AD mice, it was observed that the microglia processes directly contacted Aβ plaques [27]. In addition, in the retinal sections of APP/PS1 transgenic mice, Aβ deposits were observed in the NFL and around the retinal ganglion cells (RGCs), accompanied by a population of F4/80 microglial cells surrounding the RGCs. These deposits increased with age and promoted the overexpression of MPC-1 in the RGCs, which is an attractant of microglial cells [28].

In the control animals, most of the microglial cells were arranged parallel to the retinal surface in a regular plexus in the OPL and IPL. However, in 3xTg-AD animals, in some retinal areas, microglial cells changed their orientation by locating perpendicularly to the retinal surface, placing most of their somas in nuclear layers and sending their processes to the nearest microglial plexuses, thereby connecting them. Cell displacement and process reorientation towards an injury site are early features of microglial activation [44,45]. The microglial soma displacement towards the nuclear layers could explain the thickening in some areas of the retinal nuclear layers and the parallel thinning in plexiform layers observed by OCT using a new method for the segmentation of retinal layers in patients with mild AD [11,46]. In addition, it has been postulated that the vertical arrangement of microglial cells, like that of Müller cells, could assist in the distribution of signaling between different microglial plexuses to communicate to the rest of the retina where damage has occurred [6,47]. Therefore, although Aβ deposits were located in a certain sector of the retinal layer and could generate neuronal apoptosis only in that specific area, microglial activation could spread through the retinal layers and could induce neuronal death after its chronic activation.

Recent studies show that chronic microglial activation can be associated with deficits in the brain’s energy metabolism [48,49]. There is increasing evidence that energy metabolism and inflammation could be related and that mitochondrial energy could play an important role [50]. During the progression of AD, the microglia may initially assume a useful role to subsequently adopt a dysfunctional phenotype [51]. Thus, acute microglial activation leads to a decrease in the accumulation of Aβ by increasing its phagocytosis [41]. However, chronic microglial activation triggers different proinflammatory cascades that promote neurotoxicity and synapse loss [41]. This chronic activation may be favored by the increase in H_2_O_2_ that is generated without control by altered mitochondria [50,52]. This molecule can facilitate, on the one hand, the activation of NFκβ, which initiates and increases the expression of inflammatory genes and, on the other, the activation of inflammasome, which promotes the release of IL-1 and triggers the pyroptosis [50,53] pathways. In addition, activated microglia, unlike neurons, can collect large amounts of energy substrates for metabolism (glucose, fatty acids, and glutamine) [51]. Glutamine within mitochondria is transformed into glutamate and NH4^+^, which are neurotoxic and can contribute to neuronal death in AD [49].

In our study, we found that both types of microglial activation, acute and chronic (mentioned above), can coexist in the retinas of transgenic animals. Phagocytic amoeboid microglia, found in areas of microglial clustering, likely play a role in the cleaning of Aβ. However, the rest of the microglial cells, which also show features of activation (such as an increased cell body area, retraction of processes, changes in the orientation of the cell body, and an increase in their number), might undergo a more chronic activation. In the 3xTg-AD model, during the pre-symptomatic stage of AD, retinal microglia cells expressed anti-inflammatory neuroprotective genes (Ym1 and DC206). Nevertheless, during disease progression, the anti-inflammatory profile changed to a pro-inflammatory one, allowing potentially neurotoxic genes to be overexpressed (iNOS, IL-1β) [27]. Therefore, chronic activation of the microglia, with a constant release of proinflammatory factors, could cause a decrease in both Aβ phagocytosis and neurotoxicity, which would lead to neuronal death [35]. 

In conclusion, in the 3xTg-AD model, microglial cells showed several signs of activation, such as an increased number and soma size and the retraction and reorientation of their processes, as well as grouped cells forming rows or circular areas and changes in the cells’ locations from parallel to perpendicular to the retinal surface. These morphological changes could demonstrate that a neuroinflammatory process takes place in this model of AD. Based on this, more studies are needed to investigate whether anti-inflammatory agents or other molecules and non-pharmacological strategies could play a protective role on retinal changes in AD.

## 4. Materials and Methods

### 4.1. Animals and Ethics

Experiments were performed on 3xTg-AD mice (16 months old) harboring three human mutant genes (presenilin 1 (PS1_M146V_), amyloid precursor protein (APP_SWE_), and tau (Tau_P301L_)) and on age-matched wild type animals (WT: C57BL6/129S background) [23]. The animals were maintained at 22 ± 1 °C, 68% relative humidity, on a 12 h light/12 h dark cycle (light intensity range: 9–24 lux), with access to water and food ad libitum. All procedures involving animals were approved by the Animal Welfare Committee (ORBEA: 0421/000/000/2015. 11/20/2015) of the Coimbra Institute for Clinical and Biomedical Research (iCBR), Faculty of Medicine, University of Coimbra. The animal experiments were conducted in accordance with the European Community directive guidelines for the use of animals in the laboratory (2010/63/EU), transposed into Portuguese law in 2013 (Decreto-Lei 113/2013), and were in agreement with the Association for Research in Vision and Ophthalmology (ARVO) statement for animal use. All procedures minimized the number of animals used and their suffering.

### 4.2. Experimental Groups

Two groups of mice were used for this study: an age-matched control (WT, *n* = 8) group and a 3xTg-AD group (*n* = 8). Only the left eyes of the animals were used in our study.

### 4.3. Immunohistochemistry

Mice were anesthetized with ketamine (90 mg/kg) and xylazine (10 mg/kg) administered by intraperitoneal injection and were then transcardially perfused through the ascending aorta with 0.1 M phosphate buffer saline (PBS: 137 mM NaCl, 2.7 mM KCl, 10 mM Na_2_HPO_4_, and 1.8 KH_2_PO_4_; pH 7.4) followed by 4% paraformaldehyde (PFA) in 0.1 M PBS. 

After perfusion fixation, a stitch was made on the upper eyelid to maintain eye orientation. In addition, the nasal caruncle and the insertion of the rectus muscle were used as complementary orientation markers [47]. Later, the eyes were post-fixed for two hours in 4% PFA and kept in 0.1 M PBS. Then, the mice’s retinas were separated, the vitreous humor was eliminated by vitrectomy, and, finally, the retinas were processed as retinal whole-mounts [54].

Microglial activation was evaluated through cell morphology using an antibody against ionized calcium binding adaptor molecule 1 (Iba-1), which revealed the morphological features of the microglia [55]. Retinal whole-mounts were immunostained with rabbit anti-Iba-1 (Wako, Osaka, Japan) in a 1:500 dilution, followed by a secondary antibody, goat anti-rabbit Alexa Fluor 488 (Invitrogen, Paisley, United Kingdom), in a 1:500 dilution, as previously described [47].

The nuclei of the different retinal cells were stained with DAPI in a 1:1000 dilution, which helped to position the retinal thickness [56].

A negative control was always used to validate whether the secondary antibody reacted only with its respective primary antibody. The retinas were studied and photographed with an ApoTome device (Carl Zeiss, Munich, Germany) and with a digital high-resolution camera (Cool-SNAP Photometrics, Tucson, AZ, USA) coupled to a fluorescence microscope (Axioplan 2 Imaging Microscope Carl Zeiss, Munich, Germany), as previously described [47]. The microscope used appropriate filters for the fluorescence-emission spectra of Alexa fluor 488 (Filter set 10, Zeiss) and DAPI (Filter set 49, Zeiss). The ApoTome using the ‘structured illumination’ method allowed conventional microscopy to generate optical sections through the sample and thereby improve contrast and resolution along the optical axis. The acquired z-stacks were studied in Axiovision version 4.2 (Carl Zeiss, Munich, Germany) via the Inside 4D (3D View) tool in order to perform a cut-view analysis. A cut-view is a reconstruction of the xz and yz planes of the z-stack, which is generated by software and allows visualization through the depth of the acquired z-stack. Figure preparation was made with Adobe Photoshop CS3 Extended 10.0 (Adobe Systems, Inc., San Jose, CA, USA) [57].

### 4.4. Quantitative Retinal Analysis 

In order to analyze signs of the activation of microglial cells, we counted the following: i) The number of microglial cells in the outer plexiform layer (OPL) and in the inner retinal layer complex (ILC), constituted by the inner plexiform layer (IPL) and the nerve fiber layer–ganglion cell layer (NFL–GCL). The closeness of the Iba-1^+^ cells in the IPL and in the GCL-NFL made it difficult to differentiate the retinal microglial plexus located in each layer; therefore, we analyzed the two layers jointly. ii) The cell body area of the Iba-1^+^ cells in the OPL and ILC. These quantifications were performed in the retinal whole-mounts of naïve (*n* = 8) and 3xTg-AD eyes (*n* = 8) following similar methods to those previously described by us [58]. Retinal areas were photographed with an objective 20x/0.8 N.A. PLAN-APOCHROMAT (Carl Zeiss, Munich, Germany) at 20×, which represents an area of 0.1502 mm^2^ per field. As previously described by our group [57], each retinal wholemount was analyzed using the motorized stage of the microscope to scan the entire preparation along the *x*, *y*, and *z* axes. Cellular components in the same *xz* plane were considered to lie in the same focal plane. The microscope allows one to capture serial images on the *z*-axis (*Z*-stack). Therefore, when we begin to visualize the microglial cells, serial micrographs were made on the *z*-axis every 2 microns until the cell was no longer visible. All images thus captured corresponded to the same *xy* plane position and only changed focus in the *z*-axis. Then, we used the axiovision “extended focus” tool, whereby the serial images of the *Z*-stack were converted to a single sharp image. In this way, the extended focus module combines different “in focus” parts of each serial image by creating a single image with a greater depth of field, allowing one to visualize the entire cell and showing the maximum cell diameter and the total cell body area. This combined image created by the “extended focus” tool was used to quantify the microglial cell number and the microglial cell body area. Using this methodology, in each retinal wholemount, three equivalent areas of the retina, situated at specific distances of the optic disc in all quadrants of the retina (superior, inferior, nasal, and temporal), were photographed. In total, 12 areas were photographed per each layer (OPL, ICL), which constituted 24 images per retina. Therefore, 192 images for the 3xTgAD group and 192 images for WT group were analyzed. In addition, the means of images in every *z* stack in the OPL were 5.33 ± 0.52 for the control eyes and 4.83 ± 0.75 for AD eyes, and the means in the ICL were 6.17 ± 0.75 for the control eyes and 6.17 ± 1.17 for AD eyes.

### 4.5. Quantification of Iba-1^+^ Cells in the OPL and the ILC 

For the Iba-1^+^ cell number quantification in the different areas imaged, we used a manual counting tool included in the AxioVision Release 4.8.2 software (Zeiss, Germany) (“Interactive Measurement”), thus allowing us to calculate the average number of Iba-1^+^ cells by retinal area (0.1502 mm^2^).

### 4.6. Cell Body Area of Iba-1^+^ Cells in the OPL and ILC

In the same retinal areas selected for counting the number of Iba-1^+^ cells, the contour of the cell bodies of three microglial cells were delimited manually, to determinate their area (in μm^2^), using the “Interactive Measurement” tool included in the AxioVision Release 4.8.2 software (Zeiss, Germany). A total of 576 measurements were made for each study group (3xTgAD and WT)

### 4.7. Statistical Analysis

The statistical analysis was undertaken using IBM SPSS Statistics 25 (comprehensive statistical software; SPSS Inc©, Chicago, IL, USA). The statistical data are presented as the mean ± standard deviation (SD). Statistical analyses were carried out with a Mann–Whitney U test (unpaired data) to identify differences among the two study groups, as follows: (i) Iba-1^+^ cell number in the OPL and ILC; (ii) cell body area of Iba-1^+^ cells in the OPL and ILC. A statistical difference was considered at *p* < 0.05.

## Abbreviations

AD	Alzheimer’s disease
GCL	Ganglion cell layer
IPL	Inner plexiform layer
ILC	Inner retinal layer complex
Iba-1	Ionized calcium binding adaptor molecule 1
NFL	Nerve fiber layer
OCT	Optical Coherence Tomography
OPL	Outer plexiform layer
PFA	Paraformaldehyde
PBS	Phosphate buffer saline
PNWs	Post-natal weeks
Aβ	Protein amyloid-β
RGCs	Retinal ganglion cells
WT	Wild type
